# When the Evidence Is Incorrect: an Exploration of What Happens When Interviewers Unwittingly Present Inaccurate Information in Interviews with Suspects

**DOI:** 10.1007/s11896-021-09494-3

**Published:** 2021-12-03

**Authors:** Martijn van Beek, Ray Bull, Melissa Chen

**Affiliations:** 1 Police Academy of the Netherlands, Apeldoorn, The Netherlands; 2 University of Derby, Derby, UK; 3 Dutch National Police, Zwolle, The Netherlands

**Keywords:** Investigative interviewing, Interrogation, Police, Suspects, Information, Strategic use of evidence, Bias

## Abstract

Skillfully presenting evidence/information to suspects is one of the few interviewing techniques that increases the likelihood of guilty suspects providing information or making a confession, without making innocent ones do so as well. It is important that this evidence/information is correct, since deliberately disclosing incorrect evidence poses some risks. Also, in real-life interviews, police interviewers may unwittingly disclose incorrect evidence, for example when a witness was mistaken and provided the police with incorrect information. The present study examined the behavior of fifty police interviewers in interviews with “suspects” of a scripted crime: what is their response when the interviewees try to explain to them that some of the evidence/information just disclosed by them is incorrect? Eleven interviewers responded adaptively (by actively picking up on this new information), 35 responded in a neutral way and four responded maladaptively (by discrediting the interviewee’s claim). Experience and a full interview training had a significant negative relationship with adaptiveness. These results indicate that, when preparing and conducting interviews with suspects, greater awareness is needed of the possibility that some of the evidence/information that is to be disclosed could be incorrect, and therefore it is crucial that suspects’ responses which suggest such may be the case are taken into account.

## Introduction

Skillfully presenting evidence/information to suspects is one of the few interviewing techniques that increases the likelihood of detecting deceptive suspects (e.g., Sandham et al. 2020) and of guilty suspects providing information or making a confession, without making innocent ones do so as well (Cabbell et al. 2020; Walsh and Bull 2015). It is important, however, that this evidence/information is correct, since presenting – fabricated – incorrect evidence/information (i) reduces the likelihood of guilty suspects (who often have awareness of relevant information) making a confession (Kebbell and Daniels 2006), (ii) is related to innocent suspects making false confessions (Cabbell et al. 2020; Gudjonsson 2003; Kassin and Kiechel 1996) and (iii) is in some countries (e.g., England and Wales, The Netherlands and Norway) counter to investigator training.

However, no publications seem to have examined what happens when interviewers present evidence/information *without being aware* that some of the information is actually incorrect. Given that sources of information, such as eyewitnesses, may make errors (Lindsay et al. 2007; Toglia et al. 2007), this is a topic that requires research. The present study therefore examines the feedback/response that police interviewers provide to suspects when such suspects claim information regarding a statement of an important witness that was just disclosed to them is incorrect. That is, to what extent does the interviewers’ response/feedback reflect that the interviewers are capable of picking up on what the suspect has said, which is in contrast with the previously received witness evidence/information?

The interviewing of suspects is a topic that is receiving increasing research interest. Whereas it largely began with a focus upon factors related to the phenomenon of false confessions (Gudjonsson 2003; Kassin and Gudjonsson 2004), such as risky interrogation techniques and trait-and-state factors within the interviewee (e.g., compliance, suggestibility, sleep deprivation), more recently a substantial move was made to establish sound, evidence-based interviewing methods (e.g., Alison et al 2021; Bull 2019,2013; Bull and Rachlew 2019; Vrij and Granhag 2012; Walsh and Bull 2012) that seek relevant information. Hartwig et al. (2016) refer to these new methods as being second and third school strategies. In their categorization, first school strategies are the risky and/or unethical strategies that aim at extracting a confession using either physically or psychologically coercive methods. Second school strategies are grounded in theories of human communication, conversation management and the psychology of memory and aim at collecting an accurate and full picture of what happened, without making much distinction between interviewing witnesses or suspects. Third school strategies share the goals and purposes of second school strategies, but go beyond these by taking into account how principles of cognitive and social psychology may influence the strategic mindset both of the interviewer and the suspect.

A key element in these third school strategies is the strategic disclosure of evidence/information (Hartwig and Granhag 2015). In most real-life situations criminal investigators already have collected information that implicates the suspect to some extent before they start interviewing him/her (Hartwig et al. 2006; Hoekendijk and van Beek 2015). Disclosure strategies (Bull 2014; Oleszkiewicz and Watson 2020) address the questions of when and how interviewers should present the suspect with this already collected information. A growing body of research has found that a strategy of late or gradual disclosure of aspects of the evidence/information seems to outperform early or no disclosure in terms of being able to collect further relevant information from suspects (Walsh and Bull 2015), to corroborate on existing information (Tekin et al. 2016), to evaluate the veracity of statements made by the suspect (Sandham et al. 2020; Dando et al. 2015; Dando and Bull 2011), to assess verbal cues to deceit (Hartwig et al. 2005), to obtain and assess statement-evidence inconsistencies (Clemens et al. 2011; Oleszkiewicz and Watson 2020), and to obtain admissions (Tekin et al. 2015) or confessions (Bull and Soukara 2010). Of course, the law in many countries requires investigators to give an early explanation of why a person is being suspected, but this rarely requires all information to be disclosed and thus leaves room for a strategic disclosure of further information.

According to Srivatsav (2019), one effect of such strategic disclosure of evidence can be to alter the suspect’s perception of what the interviewer already knows and, as a result, how this may influence the suspect’s counter-strategies. Such shifts in counter-strategy may lead to new information being revealed by the suspect, or to new clues that are helpful to assess the veracity of the information given, this being the case because guilty and innocent suspects tend to differ in their counter-strategies (Hartwig and Granhag 2015). However, we argue that an implicit underlying assumption of these strategic disclosure models is that the evidence/information that is to be disclosed in the interview is correct and thus makes the suspect aware that the interviewer holds some relevant knowledge about the event(s) being investigated. Hartwig and Granhag (2015), for example, advice interviewers to *assess* the available information in regard to its *evidential value* while preparing for the interview and they posit the (guilty) suspect will have formed a hypothesis about the amount and sort of *incriminating information* the police might have available. Furthermore, deliberately disclosing incorrect information may provoke false confessions from innocent suspects if it is convincing false information, and is therefore forbidden in many jurisdictions, whereas guilty suspects may think the police “have nothing” on them.

In experimental studies, it is not a problem to be sure that the information is correct: participants perform a staged crime, what evidence is to be collected is scripted (e.g., a confederate being a witness, a camera recording a critical part of the “crime”) and thus the *ground truth* is known. In real-life investigations the ground truth is unknown and some of the information/evidence may be ambiguous (i.e., it may well fit different scenarios, either incriminating or exonerating the suspect) or incorrect. For example, criminal investigators often have to rely upon (i) statements of witnesses, who could be mistaken (Lindsay et al. 2007; Toglia et al. 2007) or could have a range of different reasons to make false allegations (de Zutter 2017; McNamara et al. 2012), or upon (ii) forensic evidence, that might be the outcome of false positive errors (Kassin et al. 2013) or other misinterpretations (Dror et al. 2006; Dror and Hampikian 2011; Huang and Bull 2021). The University of Exeter’s Miscarriages of Justice Registry, created by the Evidence-Based Justice Lab (2021), contains 398 cases of wrongful convictions in the UK and Wales between 1970 and 2016. In 155 cases (39%) a witness testimony proved incorrect, and 75 cases (19%) involved false or misleading forensic evidence. Given these numbers, it may fairly often happen in real-life interviews that interviewers unwittingly disclose one or more pieces of incorrect information to the suspect. Furthermore, when preparing for an interview and assessing the available information, investigators may have a tendency to focus on selecting incriminating evidence/information because of a guilt bias (Ask and Granhag 2005; Marksteiner et al. 2011). This may lead them to overlook the possibility that the incriminating information actually is incorrect and therefore not incriminating after all. Smith and Bull (2013), for example, found that their sample of nearly 400 criminal investigators from several countries tended to perceive forensic evidence as strong evidence and claimed that having forensic evidence available had an impact on their interview strategy. Yet, most of them indicated that they were not trained to interpret forensic evidence. This apparent lack of procedures and guidelines is, next to a potential guilt bias, another reason to assume that it is plausible that information may be misinterpreted and therefore used incorrectly in interviews.

For strategic interviewing methods to be effective and without risk in field settings implies that interviewers applying such methods must be sufficiently open-minded while preparing, conducting and evaluating a suspect interview to withstand their potential biases (Boyle and Vullierme 2018; Bull 2018; College of Policing 2021; Kleinman, in Snook et al. 2020a; Leahy-Harland and Bull 2016; Meissner et al. 2015). This means that it is expected of interviewers that during the interview, they will be able to keep in mind that information they disclose to suspects may be incorrect.

However, as Ask and Alison (2017) explain, solving crime is a complex information processing task and the human brain’s capacity to process information is limited. These contradicting factors will “restrict the possibilities of investigators remaining open-minded, impartial and thorough at all times” (Ask and Alison 2017, p. 37). This may be especially hazardous when investigators change (too soon) from a hypothesis-testing mode (taking into account all potential scenarios) to a case-building mode (collecting evidence to prove the case) (Ask and Alison 2017). However, this shift from deliberative thinking to implemental thinking (Gollwitzer et al. 1990) occurs frequently—and is even “mandatory”—in criminal investigations, for it is necessary to be able to prosecute a likely guilty suspect (Ask and Alison 2017; Fahsing and Ask 2013). According to a sample of 35 experienced Norwegian and English homicide investigators, the decision to identify, arrest, or charge a suspect is a typical event that will trigger such a shift (Fahsing and Ask 2013). In some studies, investigative experience has been found to be a mediating factor (Fahsing and Ask 2015; Dando and Ormerod 2017), indicating that experienced investigators are more able to withstand confirmation bias compared to novices. In other studies, however, this mediating factor was not present (Fahsing and Ask 2015). These findings indicate that it is likely that criminal investigators are already in case-building mode from the beginning of the first interview with a suspect, which is not in line with what is advised in for example the “PEACE” method of investigative interviewing, introduced in 1992 by police in England and Wales (Milne and Bull 1999).

Besides being open-minded, PEACE also advocates a rapport-based approach; with rapport referring to a working relationship between interviewer and interviewee that is in line with a more humane interviewing technique, which improves investigative outcomes (Bull and Baker 2020; Vallano and Schreiber Compo 2015). Holmberg and Christianson (2002) innovatively found that their sample of men convicted of murder or sexual crimes did not only prefer such a humanitarian, rapport-based approach over a dominant approach, this humanitarian style was also related to admissions whereas the dominant style was related to denials.

**Fig. 1:**
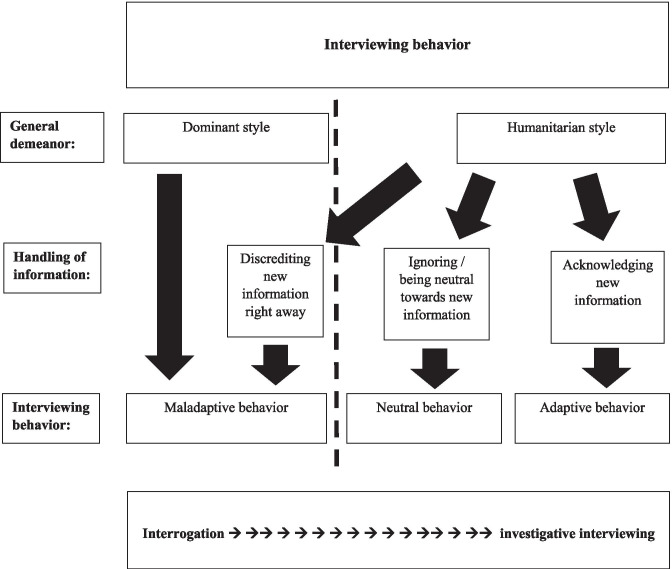
Interviewing behavior displayed by interviewers and their handling of new information

In his commentary in Snook et al. (2020a), Kleinman proposes his model of “feeling–thinking–acting,” in which the notions of (i) strategic interviewing, (ii) information management, and (iii) rapport-based interviewing come together. Kleinman argues that while (i) empathy (“feeling”) is the foundation of “operational accord” (his wording for rapport—see Bull and Baker 2020), (ii) interviewers are faced with a “multifaceted sense making task” in which they have to avoid cognitive biases by deliberately applying “debiasing strategies” (“thinking”), and therefore, (iii) interviewers must have the ability to adapt behaviorally and cognitively in response to fluctuating scenarios (“acting”). “The adaptive interrogator is one who is relatively comfortable with ambiguity and thus more readily able to find meaning within confusing circumstances,” Kleinman writes in Snook et al. (2020a, p. 6). We therefore suggest that although using a humanitarian approach is a basic ingredient to steer clear from risky interviewing behavior (Surmon-Böhr et al. 2020), really good, skillful, unbiased interviewing requires something more: the capability of the interviewer to pick up on relevant new and possibly contradictory information that may emerge within an interview. Picking up on such information, which may conflict with already known or assumed information, requires skills like critical thinking, adaptability (Mount and Mazerolle 2020) and responsiveness towards the suspect (Bull and Soukara 2010). Interviewers who directly discredit such information as “not true” on the basis of the already known or assumed information they received earlier on, may be classified as displaying *maladaptive* behavior, even when they are doing so whilst maintaining rapport. In this case, they may appear to behave in a “humanitarian” style, but they are only doing so on a more superficial level (see e.g., Adams-Quackenbush et al. 2019, for a case study on this). Conversely, interviewers who explore this new information within a humanitarian style and provide the suspect with relevant feedback in relation to it (e.g., by taking into account or testing this new information), display *adaptive* behavior. Interviewers who do not make clear whether they have taken in the new information or do not show to the suspect what they will do with it, display *neutral* behavior. In the words of Kleinman’s model: these interviewers may perhaps have reached the level of thinking, but do not convert this thinking into acting. Such neutral behavior may keep interviewees “in the dark” as to whether they are really being listened to. This approach may eventually hamper rapport and disrupt the flow of information. The flow chart in Fig. 1 shows a conceptual model that integrates the above mentioned concepts for the present study.

## Aim of the Study

The aim of the present study is to explore what happens when interviewers unwittingly present incorrect information to suspects when they are strategically disclosing evidence. We therefore created a design wherein interviewers had to disclose several pieces of potentially incriminating information to suspects (of a staged crime). One of those pieces was a witness’ allegation that proved to be untruthful, but that was not known to the police at the time of the interview—and also could not be known at that time.

Rather than focusing on the interviewees’ responses when being presented with this incorrect information, we focused on the interviewers’ reactions to cues in the interviewees’ responses indicating that this presented information may actually be incorrect. We assessed whether the interviewers seemed to discredit the interviewees’ responses right away (*maladaptive* behavior), acted neutral or indifferent towards these cues (*neutral* behavior) or displayed open-mindedness by actively picking up on this new information (*adaptive* behavior).

Since real-life interviews have increasingly been recorded in recent decades, observing what happens in these interviews has now become possible (Milne et al. 2008). In general, studies of real-life interviews report mixed findings in regard to interview quality, even in countries where relatively more effort has been put into interview training such as the UK (e.g., Clarke and Milne 2016; Griffiths and Milne 2006; Walsh and Bull 2010). Walsh et al. (2016) concluded in their commentary on international developments in interviewing and interrogation that British police interviewers are nowadays able to avoid oppressive and confession-centered techniques, but still struggle with the skills of rapport-building, summarizing and developing effective strategies. In a similar vein, Akca et al. (2021) found that training in evidence based investigative interviewing models has some positive impact on *basic* interviewing skills, but far less on *cognitively more demanding* skills. Verhoeven and Duinhof (2017) found that interviewers in the country of the present study made use of 76 different interviewing techniques, displaying both (i) advocated, investigative interviewing techniques (e.g., encouraging the suspect to tell his/her side of the story, asking open questions, building rapport, explaining the situation, informing the suspect of his/her rights) and (ii) more coercive interviewing techniques, especially in combination with disclosing evidence to less or non-cooperative suspects (e.g., asking leading questions, disputing the information provided by the suspect, emphasizing the interviewer’s authority, making accusatory comments).

Given these findings, in this present study we attempted to explore the reactions of experienced police interviewers when faced with interviewees suspected of a crime, who dispute or correct some incorrect information that the interviewer has disclosed in the interview. The interviewers’ job *requires* that they behave adaptively, but given the complexity of information management tasks and the risk of them having already shifted to a case-building mind-set, it is expected that many of them will *not* behave adaptively.

## Method

### Overview

In this study, fifty interviews were observed wherein experienced police interviewers interviewed suspects of a staged crime. We here only analyze those parts were the incorrect information at stake—the witness’ allegation—was being discussed. The selected interviews are part of a greater sample that is being collected to address another research question; however, the accumulation of this greater sample has been temporarily interrupted because of COVID-19 restrictions in the relevant country. In the greater sample some of the interviewers performed more than one interview. For the present study, we examined only the first interview of each unique interviewer.

### Participants

A total of fifty vocational education students participated as a suspect in this study. Their mean age was eighteen years (mean = 18.48 years, SD = 1.072 years; range = 17 to 21 years; missing values = 2). This age corresponds with the peak age for crime commission (Hirschi and Gottfredson 1983). Sixteen were male and 34 female. All interviewees were enrolled in a safety and security education program at training centers having a covenant with the relevant Police Academy. When these students pass their final exam, they are automatically eligible to apply for a job within the police organization. When accepted they receive an accelerated police training. These students were recruited for this study by contacting their schools. It was then an individual and free choice to participate or not.

The interviews were conducted by 24 male and 26 female police interviewers, all receiving or having received at least the basic version (modules 1–5 and A) of the professional training in interviewing (PTI) of the Police Academy of the relevant country (see van Beek and Hoekendijk 2016). They were recruited via snowball sampling in the network of the first author. The interviewers can be categorized into four sub-groups: having received the Basic PTI (modules 1–5 and A^1^)—18 interviewers; having received the Full PTI (also received modules 6–8 and B of the PTI)—21 interviewers; having received the IVS (a follow-up training in Interviewing Vulnerable Suspects after having received the Full PTI, see van Beek and Hoekendijk 2016)—7 interviewers; and having a master’s degree in criminal investigation (MCI) degree—4 interviewers. Investigators with a master’s degree in criminal investigation are qualified at level 7 of the European Qualification Framework – EQF (Europass 2021), whereas the other investigators in the study are qualified at level EQF 4.

The interviewers’ mean age was 45 years (mean = 45.11 years, SD = 8.17 years; range = 30 to 63 years; missing values = 3). Their interviewing experience was on average nearly fifteen years; however, there were large individual differences in the amount of experience (mean = 14.98 years, SD = 8.54 years; range = 2 to 43 years; median = 12.50 years; mode = 10 years).

### Procedure

The collected sample contained 32 interviews with guilty “suspects” and 18 interviews with innocent “suspects.” The innocents showed “suspicious” behavior that aligned with the correctly disclosed pieces of evidence/information being true, but were innocent of the crime itself. Yet, in regard to answering the current research question, we did not distinguish between guilty and innocent suspects as the conditions in regard to the incorrect information at stake were similar for both groups.

The suspects were instructed to take their head teacher’s new and expensive smartphone from the teacher’s office (guilty ones) or to take a look at it and let it slip out of their hands while in the office (innocent ones), and did so by following a provided script. The teacher acted actually as a confederate. Evidence was collected in the form of (i) witness statements (another teacher and a befriended classmate of the participant also acted as confederates and gave a scripted witness statement to the police), (ii) computer log data (after taking the phone the participant had to search the internet to find out the price of a second-hand smartphone similar to the teacher’s phone), and (iii) a “still” from the school’s video surveillance system showing the participant leaving the school at the day of the crime.

The interviewers received the aforementioned pieces of information and also a witness statement from a schoolmate of the participant. They were informed that this schoolmate came to the police station spontaneously and told the police that in the morning break at school the day after the crime the participant had asked him whether he knew someone who was interested in buying a second-hand phone similar to the teacher’s missing phone.

A few days after the crime, all the “suspects” were invited to come to the local police station to be interviewed. The interviewers were instructed to conduct these interviews in the professional, humanitarian way that had been taught to them in their interview training. They interviewed their “suspects” with the help of an interview plan that was written according to the principles of gradually and strategically disclosing evidence. A senior interview trainer of the relevant Police Academy wrote this interview plan (see Appendix), which contained all the aforementioned pieces of information. The interviewers were asked to keep the plan in mind: asking some follow-up questions on the same topic was explicitly allowed when deemed necessary, as long as they eventually would return to the plan to make sure all topics were covered and in the prescribed order.

The interviewers and the author of the interview plan did not know that the schoolmate-witness had lied to the police about talking to the suspect during morning break. This chat actually had never happened. When being questioned about this schoolmate’s statement, all participating interviewees were instructed to:

- tell that they once had a quarrel with this schoolmate,
- claim he is someone they do not trust, because he is “always telling stories”,
- deny they spoke to him during that particular morning break,
- provide a checkable alibi for what they did instead (e.g., having a chat with some other classmates^2^).

Each interviewer interviewed one interviewee, pairs were matched at random and all interviews were recorded audio-visually. All participants, interviewers and interviewees, were informed that they would participate in a study regarding the effectiveness of interview strategies. They were unaware of the exact purposes of the study. They received no incentives for participating. However, most were able to participate under working/studying time. They were all allowed to withdraw at any given moment. Ethical approval was obtained from the Ethics Committee of the relevant university (code ETH1819-0048).

### Analysis

A first general analysis was performed to ensure the “suspects” did follow up on the instructions they had received in regard to the incorrect piece of information. All interviewees indeed provided the information as instructed: they told the interviewers that the witness was in their view not a reliable person, that they had once had a quarrel, that the meeting and the conversation in the morning break did not take place, and that they were at that time with other friends/classmates who could confirm their alibi. However, they did differ in exactly how unreliable they found the witness. Their answers ranged from “I don’t really know him that well, so I cannot say much about how reliable he is” to “he is a very unreliable person,” the latter often accompanied with examples of unreliable behavior. Nearly all interviewees were willing to provide information. This does not mean that they always gave complete and honest answers, but they did at least engage appropriately in the conversation with the interviewers. Only two interviewees were found to be quite obstinate. It was decided to leave these interviews in the sample for further analysis.

In this second step, two raters (the first and third author of this paper) independently observed and coded the interviewer behavior for all parts of the interviews that related to the incorrect piece of information. The interviewers’ behavior was scored on a 5-point Likert scale as being maladaptive, moderately maladaptive, neutral, moderately adaptive or adaptive, with “maladaptive” meaning that the interviewer displayed some behavior that indicated he/she was more inclined to believe the witness who had lied than the suspect (who told the truth in regard to this piece of information), and “adaptive” meaning that the interviewer displayed some behavior that indicated he/she took into account the new, contradictory information provided by the interviewee (see Table 1 for further detail).

**Table 1:** Scoring of interviewers’ behavior

Score	Meaning	Examples of behavior	Interview examples	Outcome
− 2	Utterances of the interviewer indicate (s)he is favoring the statement made by DS over the information provided by the suspect	Interviewer states directly (s)he doesn’t believe the interviewee, but does believe DS Interviewer discredits the interviewee’s response and comes up with alternative theories to explain why DS is right and the interviewee is wrong	“You say X, DS said Y, so one of you is lying and there is no reason for DS to lie. Come on.” “Well, I think you are confusing this break with another break.”	Maladaptive
− 1	Utterances of the interviewer indicate (s)he is to some extent favoring the statement made by DS over the information provided by the suspect	Interviewer states (s)he is inclined to believe DS over interviewee Interviewer doesn’t seem to listen to interviewee’s response to the challenge, but instead repeatedly challenges the interviewee with the statement of DS Interviewer suggests there might be alternative theories to explain why DS could be right and the interviewee could be wrong	IR: “DS told us he spoke to you that morning break.” IE: “I haven’t met him. I was with my friends, X and Y.” IR: “Are you sure, since DS told us he spoke to you that morning break.”	Moderately maladaptive
0	Utterances of the interviewer indicate that the interviewer either (i) strictly follows the instruction to stick to the interview plan, and/or (ii) doesn’t put more weight on either the interviewee’s version of events nor DS’s version	Interviewer sticks to remarks that are written down in the interview plan Interviewer is being neutral consistently During the interview the interviewer does not show whether (s)he (i) favors the statement of DS over the interviewee’s responses or (ii) incorporates the new information in the investigation	IR: “DS told us X.” IE: “But that didn’t happen.” IR does not summarize, but continues with the next topic OR “Okay, you say X and DS said Y. I will write my report and then it is up to the prosecutor to decide.”	Neutral
1	Utterances of the interviewer indicate (s)he is to some extent incorporating the new information provided by the suspect into the investigation	Interviewer states (s)he is inclined to check the new information Interviewer leaves out the information provided by DS at some point in the interview	IR:“Okay, so you say DS is untrustworthy and you had an argument. However, he said X and you say Y. Of course, we will need to investigate this further.” OR IR: “Okay, so we have Piece X, Piece Y, and the statement of DS.” IE: “But what DS said it isn’t true.” IR: “Once again, we have Piece X and Piece Y. What is your response to these?” [Interviewer thus drops the statement of DS]	Moderately adaptive
2	Utterances of the interviewer indicate (s)he is incorporating the new information provided by the suspect into the investigation	Interviewer states the new information will be investigated Interviewer clearly and precisely summarizes IE’s responses (and thereby securing this information for upcoming investigations and prosecution decisions)	IR: “Okay, so he says X and you say Y. We will need to investigate this. Who was this friend again that you said could confirm your story?” OR IR: “You told me X when DS said Y. However, you told me to also take into account that DS and you had an argument once and that you think he is not very reliable.”	Adaptive

IR = interviewer; IE = interviewee; DS = Dylano Schemer, the schoolmate-witness. (Please note that “schemer” does not have a double meaning in the relevant language.)

Cohen’s κ was used to determine the extent of agreement between both raters. There was good agreement between them (κ = 0.707, *p* < 0.0001). Both raters then discussed the interviews they scored differently until they had agreed upon a final score for each of these interviews.

In order to see whether they made references to the incorrect piece of information, all answers the interviewers gave to the open questions in their post-interview questionnaire were analyzed as well. Although these questions were originally developed to address manipulation checks and other research questions, the answers may reveal further information regarding the interviewers’ adaptiveness. This post-interview questionnaire contained the following open questions (often preceded by or in combination with some closed or Likert-style questions):

- When you thought the interviewee was lying to you, what where the indications to think so?
- According to you, what are indications of lying in general?
- If your assumptions regarding the potential guilt or innocence of the interviewee changed during the interview, what were the reasons for this change?
- At what moment in the interview did such a change take place?
- Was the interview plan representative or not according to you, and why?
- In retrospect, what would you have done differently in the interview, and why?
- Are there any other comments you wish to make?

The fragments that were deemed relevant to the current study were given a code that summarized its content (e.g., “other scenarios still applicable”); fragments with codes that seemed to be similar were then grouped together in subcategories with an overarching code; and finally, these subcategories were categorized as whether the fragments they contained were either an indication of adaptive or maladaptive thinking.

## Results

In a majority of the interviews, the interviewers displayed at least *neutral* behavior towards the interviewee when the incorrect evidence was being discussed: 35 of the interviewers were neutral in this regard (among them both interviewers of the two obstinate interviewees), ten were moderately adaptive and one was adaptive. The interview behavior of only three interviewers was scored as moderately maladaptive in this regard, and that of one interviewer as maladaptive. This means that in the coded fragments of the interviews 46 of the interviewers were sufficiently humanitarian in their approach. However, in order to test whether they were also able to be as adaptive as required, the outcomes were transferred into a dichotomous set of variables: all maladaptive, moderately maladaptive, and neutral scores (total = 39) were grouped into the new variable “not picked up on,” all moderately adaptive and adaptive scores (*n* = 11) were grouped into the new variable “picked up on.” A one-sample binomial test with Clopper-Pearson 95% CI then was performed on these data, which resulted in a 95% CI of 64.0% to 88.5%, *p* < 0.0001. We therefore conclude that, despite their humanitarian approach, a significant majority of the interviewers did not actively demonstrate to the interviewee that they had picked up on the new information.

In the post-interview questionnaire, fourteen interviewers made comments that were considered relevant to this study. The main finding is that eleven neutral interviewers may not have said it aloud in their interview, but were, as their comments show, still at least quite open-minded in regard to the suspect’s statements. In more detail, eight of them made the general comment that their suspect could in their opinion still be innocent (seven of these interviewees were actually guilty), and the other three mentioned that the remarks of the witness that provided the incorrect information should be subject to further investigation. Two moderately adaptive interviewers also made this latter comment. One neutral interviewer made a more maladaptive comment. This interviewer wrote that the suspect denying that the conversation with the witness had taken place was very clearly a sign of lying.

To test whether factors could be found that would predict adaptive or maladaptive interviewers’ behavior, ordinal regression tests were conducted. In the first test the dependent variable being used was adaptiveness. The independent variables were “interviewee confessed or not,” “interviewee gender,” “interviewer gender,” “interviewee guilty or innocent,” “interviewer age,” “interviewer educational level,” and “interviewer experience.” (The factors of “interviewee age” and “interviewee educational level” were not used since all participants were of a very similar age and each have the same educational level.) The model in this first test did not have a good fit: *χ*^2^(9) = 15.833, Nagelkerke *R*^2^ = 0.342, *p* = 0.070. The variable “interviewer experience” did, however, have a significant *negative* association with adaptiveness (estimate =  − 1.35, SD = 0.060, *p* = 0.025). Having followed the Full PTI also had a significant *negative* association with adaptiveness in comparison with the other professional education levels (i.e., Basic PTI, IVS and MCI): estimate =  − 3.712, SD = 1.448, *p* = 0.010.

In a second and third test, respectively, the weakest predictive variable—“interviewee guilty or innocent”—and the two weakest predictive variables—“interviewee guilty or innocent” and “interviewee confessed or did not”—were left out of the calculations. The third model does have a good fit^3^: *χ*^2^(7) = 14.493, Nagelkerke *R*^2^ = 0.317, *p* = 0.043. Also in this third model, interviewing experience has a significant negative association (estimate =  − 0.118, SD = 0.057, *p* = 0.039), as does “Full PTI” compared to the other educational levels (estimate =  − 3.700, SD = 1.391, *p* = 0.008). See Table 2 for the full results of this model.

**Table 2:** Ordinal regression model regarding adaptive or maladaptive behavior of interviewers

		Estimate	Std. error	*p* value
Threshold	Maladaptive	− 5.246	2.454	0.033*
	Moderately maladaptive	− 3.673	2.279	0.107
	Neutral	1.372	2.190	0.531
	Moderately adaptive	4.107	2.370	0.083
Location	Interviewer age	0.094	0.061	0.123
	Interviewer experience	− 0.118	0.057	0.039*
	Interviewee gender M	− 0.980	0.772	0.205
	Interviewee gender F	0		
	Interviewer gender M	− 0.960	0.850	0.259
	Interviewer gender F	0		
	Educational level MCI	− 0.480	1.487	0.747
	Educational level Basic PTI	− 1.199	1.083	0.268
	Educational level Full PTI	− 3.700	1.391	0.008*
	Educational level IVS	0		
Model fitting information: *χ*^2^(7) = 14.493, Nagelkerke *R*^2^ = 0.317, *p* = 0.043*				

^*^ *p* < 0.05

## Discussion

As was expected given their previous interview training, most of the participating interviewers were able to display sufficient humanitarian interviewing behavior within the coded fragments of their interview. Nevertheless, in line with our expectations, only a minority displayed real *adaptive* behavior, by providing feedback to the interviewee that they actively picked up that the interviewee was providing them with information that potentially shed a new light on one of the pieces of information (that actually was incorrect). However, indications were also found that some of the interviewers who did not display such adaptive behavior, did at least *notice* that the interviewee provided such information to them. Contrary to what might have been expected, *interviewer experience* and being trained at the *rather high level* of the Full PTI had a negative relationship to adaptiveness.

Adaptiveness may be regarded as displaying a set of skills that requires high expertise in the already complex cognitive task of interviewing a suspect: the interviewer has to *formulate accurate questions*, has to *listen well* to the interviewee, needs to *realize* that the interviewee is providing *relevant* new information that could *contradict* information that was received earlier, and has to *respond appropriately* to this new information. Our findings seem to be in line with earlier observations that trained interviewers may still have difficulties with skillfully performing more complex cognitive tasks (Walsh et al. 2016; Akca et al. 2021).

The negative relationships between (i) interview experience and adaptive behavior, and between (ii) the Full PTI and adaptive behavior, seem at first sight to be at odds with this, but these findings may be explained by developments in the relevant country. In the aftermath of a case in which an innocent man was convicted of murder after he had made a false confession, a major improvement program took place in 2005 (Posthumus 2005). This program led to the implementation of the PTI in 2006 (nowadays known as Full PTI). In 2013 the current, revised curriculum of the PTI was implemented, making a distinction between a basic training (Basic PTI) and an extended follow-up training that can be deferred (together: Full PTI). Furthermore, in 2017 the interviewing method taught in the PTI was revised in order to put more emphasis on exploring alternative explanations that are put forward by suspects (van Amelsvoort and Rispens 2021; Rispens et al. 2017). Taking into account that cultural changes may take a fair amount of time and that several of the Full PTI participants may have received their training years before the Basic PTI participants did, it is possible that the findings in this study in regard to more *experience* and a *Full PTI* level of education are actually confounded by factors such as having received training longer ago, having received a less modern training, and/or having been “raised” in a workplace culture that more strongly advocated “old school habits”. Unfortunately, we do not have the data to test these notions (e.g., an answer to the question of *when* the participants received their training). Developments in England and Wales, however, prove that training programs in investigative interviewing had to be revised multiple times before these became effective (Clarke and Milne 2016). Since the Full PTI is required to enter the IVS training, the interviewers at the IVS-level have received more interview training, and especially in skills that support being responsive to (the needs of vulnerable) suspects. The MCI’s, qualified at EQF 7, may have greater cognitive abilities compared to the Full PTI-participants, qualified at EQF 4, and may therefore be more equipped in handling cognitively more demanding tasks.

Our findings are relevant for theories of strategic interviewing. Two decades of research have built a firm foundation for the notion that strategically disclosing evidence to suspects fosters the gathering of reliable and often incriminating information in an ethical and effective way (Bull et al. 2019; Hartwig and Granhag 2015; Hartwig et al. 2016; Oleszkiewicz and Watson 2020; Sandham et al. 2020), and helps to obtain comprehensive accounts (Bull and Soukara 2010; Soukara 2005; Walsh and Bull 2012). Criminal investigators in a growing number of countries have been trained in several models of strategic interviewing (van Beek and Hoekendijk 2016; Clemens et al. 2019; King (2002) in Sukumar et al. 2016; Luke et al. 2016; Nilsson, personal communication, 2019, November, 27; Rachlew, personal communication, 2020, March, 3; Snook et al. 2020b). However, as stated in our introduction, the strategic interviewing research thus far has implicitly assumed that the evidence/information that is to be disclosed to the suspect is all correct information. In real-world policing, where the ground truth is—in the nature of the matter—unknown, this cannot be assumed. The present study found that only a minority of interviewers displayed really adaptive behavior when they were confronted with opposing information. Further research into the potential effects of unwittingly disclosing incorrect evidence/information to suspects is therefore required, especially since it is well established that consciously disclosing false evidence in criminal investigations can have enormous consequences on not only in what it tells guilty suspects about the strength of the interviewer’s case (Bull 2014), but also on the likelihood of innocent suspects making false confessions (Cabbell et al. 2020; Gudjonsson 2003; Kassin and Kiechel 1996). Snook et al. (2020b) explicitly warn the police to not abuse strategic interviewing by disclosing inaccurate or hypothetical information. Follow-up research could address topics such as (i) what is the critical threshold for evidence/information being accurate enough to be used safely within a model of strategic interviewing (see for example the findings of Smith and Bull 2013, regarding there currently not being much guidance on pre-interview information assessment), (ii) what corrective mechanisms need to be in place when preparing, conducting or evaluating the interview, and (iii) what happens subsequently in the investigations when a suspect contradicts the information that was collected earlier.

Picking up on such information, instead of disputing or ignoring it, could serve to construct new investigative links and thus be helpful for the police in being able to reconstruct what happened. Police interviewers need to be competent in this regard, either as a result of recruitment or training. This present study indicates that *expertise* may perhaps be more important in this regard than mere *experience*. This hypothesis will need to be further explored as well. Furthermore, we found quite a number of neutral interviewers that perhaps may not have been adaptively enough in their interview behavior, but that nevertheless proved to be—latently—open-minded in their thinking, given their comments in the questionnaire. The question of how to bridge the gap from Kleinman’s concept (in Snook et al. 2020a) of *thinking* to his concept of *acting* is therefore relevant, especially for interview trainers.

The current, pioneering study—it being the first to explore the influence of unwittingly disclosing incorrect evidence/information—has its limitations of course. First of all, the coders were not blind to the research question. However, they first scored the interviewers’ behavior independently from one another, and inter-rater reliability is good. Second, although we did explore whether during the interview interviewers would *act* when the suspect provided them with information that contradicted their previously received information (level 3 of Kleinman’s model), we did not examine in detail what the interviewers *thought* (level 2 of Kleinman’s model) when listening to the interviewee’s responses. As stated above, at least some of the interviewers seem to have noticed what the interviewees told them, but for some reason did not act upon it.

In our study we did not control for several factors that perhaps may have played a mediating role, such as interviewer factors (e.g., personality, attitudes, previous experiences) or interviewee factors (e.g., their behavior in regard to the other topics that are discussed in the interview, the tone of voice/intensity with which they claimed that the incorrect information was not true), nor did we control for factors related to the interaction between the interviewer and interviewee (e.g., how did they perceive one another). Some interviewers in the present study may have met, as a consequence, “harder conditions” compared to other interviewers, and therefore their interview behavior may have been judged maladaptive or neutral instead of adaptive. Such nuances enhance the generalizability of the findings as these factors are inherently part of a more naturalistic context and thus add value to this element of the study: after all, in real-life an interviewer has the difficult task of being adaptive to the interviewee’s responses *at all times*, no matter whether such factors are at stake or not. A limitation could be that the interviewees were *instructed* in regard of this particular part of the interview, whereas they were free in their responses in the other parts. This may have served the internal validity of this present study, but in contrast, it may have hampered the overall naturalistic conditions.

Another potential limitation is that while in real-life situations interviewers may write an interview plan themselves, in this study they worked with a provided plan and did not have much time to get familiar with this plan and the case details (the latter does happen however in real-life investigations). Working with someone else’s plan (which sometimes actually happens, especially in countries where interview advisors prepare the plan) could have been an extra cognitive demand within an already cognitively demanding task, leaving less mental space to improvise during the interview when this was required. Furthermore, the role-play setting may have given them the (false) impression that all the information they received was controlled and correct information, whereas in real-life they may be more critical in this regard. For example, our interviewers had no background information regarding the witness who made the false accusation. In real-life situations, such information might have given an indication prior to the interview to be cautious with the information provided by this witness, or to check it further before presenting it to the suspect. Launay et al. (2021) found that in mock interviews with witnesses, 22% of the questions were related to assessing the reliability of either the witness or the witness statement. The interview plan of our participants lacked such contextual information regarding the schoolmate-witness, although questions regarding the suspect’s perspective of this witness’ reliability were included in the plan and all interviewees made it clear to the interviewers that, at least to their opinion, this witness was not that credible.

A final, potential limitation is that we asked the interviewers to put in a lot of effort for what could be considered a minor crime. A minor crime was chosen in order to avoid the interviewers being presented with overly complex details or too many of them. For interviewers who normally work in high stakes or complex crime investigations (as our sample does) this might have been somewhat artificial. On the other hand, by asking criminal investigators to participate, inviting the interviewees to come over to the police station to be interviewed in interview rooms, making use of an example of incorrect information that happens relatively often (an incorrect statement of a witness) and instructing the interviewees to provide a commonly used alibi (“I was with friends”) this study contained various important naturalistic, ecologically valid elements.

In sum, it can be concluded that within the paradigm of strategic interviewing it is important that police interviewers are aware of the possibility of some of the evidence/information they strategically wish to present to the suspect actually being incorrect. This is due to a variety of reasons that are inherently part of the processes within criminal investigations. First and foremost that the ground truth is not known: in fact, a thorough reconstruction of what is mostly likely to be true—an “inference to the best explanation” beyond a point of “reasonable doubt”—is what is being asked of an investigator (Van Koppen and Mackor 2019). This means that police interviewers should at all times listen carefully to the suspect’s responses, so any indications of the presented evidence/information being potentially incorrect are detected and can be acted upon by reflecting these back to the interviewee.

## Data Availability

Data and material are available from the corresponding author upon reasonable request.

## Appendix. The interview plan

Interview steps 8, 9, 14, and 15 are relevant in regard to the incorrect piece of information.

## Footnotes

1 The Basic PTI contains modules in personal efficacy (self-reflection), the evidence matrix (a tool for assessing the information already available at the start of the interview), person-oriented interviewing (building rapport), case-oriented interviewing (discussing case-related topics with the suspect) and interviewing witnesses (modules 1–5). In Module A, interviewers prepare and conduct a real-life suspect interview under supervision. The Full PTI also contains modules in regard to (inter-)cultural awareness, psychopathology and working together with third parties (e.g., interpreter) (modules 6–8). In Module B, interviewers prepare and conduct, under supervision, a real-life suspect interview that contains such factors. See van Beek and Hoekendijk (2016) for further detail.

2 Alibis in the form of witness evidence are the most common alibis provided by suspects, but tend to have a lesser degree of believability among both law professionals and lay people compared to forensic evidence (Nieuwkamp 2018).

3 In order to test the assumption of there being no multicollinearity, a linear regression test with collinearity diagnostics was performed. VIF-values were respectively 1.047 for ‘interviewee gender’, 1.089 for ‘interviewer gender’, 1.657 for ‘interviewer age’, 1.051 for ‘interviewer educational level’ and 1.591 for ‘interviewer experience’.

## Code Availability

A custom way of coding has been used and is explained in the manuscript.
